# Barriers to Breast Cancer Screening among Diverse Cultural Groups in Melbourne, Australia

**DOI:** 10.3390/ijerph15081677

**Published:** 2018-08-07

**Authors:** Jonathan O’Hara, Crystal McPhee, Sarity Dodson, Annie Cooper, Carol Wildey, Melanie Hawkins, Alexandra Fulton, Vicki Pridmore, Victoria Cuevas, Mathew Scanlon, Patricia M. Livingston, Richard H. Osborne, Alison Beauchamp

**Affiliations:** 1 Health Systems Improvement Unit, Centre for Population Health Research, School of Health and Social Development, Deakin University, Geelong 3220, Australia; jonathan.ohara@deakin.edu.au (J.O.); crystal.mcphee@deakin.edu.au (C.M.); sdodson@hollows.org (S.D.); carol.wildey@semphn.org.au (C.W.); melanie.hawkins@deakin.edu.au (M.H.); alexandra.fulton@tgarage.com.au (A.F.); trish.livingston@deakin.edu.au (P.M.L.); richard.osborne@deakin.edu.au (R.H.O.); 2 The Fred Hollows Foundation, Melbourne 3053, Australia; 3 BreastScreen Victoria, Melbourne 3053, Australia; acooper@breastscreen.org.au (A.C.); vickip@breastscreen.org.au (V.P.); vcuevas@breastscreen.org.au (V.C.); mscanlon@breastscreen.org.au (M.S.); 4 Department of Rural Health, Monash University, Moe 3825, Australia; 5 Department of Medicine-Western Health, The University of Melbourne, St Albans 3021, Australia; 6 Australian Institute for Musculoskeletal Science (AIMSS), The University of Melbourne and Western Health, St Albans 3021, Victoria, Australia

**Keywords:** health literacy, HLQ, breast cancer, breast cancer screening, mammography, Italian, Arabic, CALD

## Abstract

This study explored the association between health literacy, barriers to breast cancer screening, and breast screening participation for women from culturally and linguistically diverse (CALD) backgrounds. English-, Arabic- and Italian-speaking women (*n* = 317) between the ages of 50 to 74 in North West Melbourne, Australia were recruited to complete a survey exploring health literacy, barriers to breast cancer screening, and self-reported screening participation. A total of 219 women (69%) reported having a breast screen within the past two years. Results revealed that health literacy was not associated with screening participation. Instead, emotional barriers were a significant factor in the self-reported uptake of screening. Three health literacy domains were related to lower emotional breast screening barriers, feeling understood and supported by healthcare providers, social support for health and understanding health information well enough to know what to do. Compared with English- and Italian-speaking women, Arabic-speaking women reported more emotional barriers to screening and greater challenges in understanding health information well enough to know what to do. Interventions that can improve breast screening participation rates should aim to reduce emotional barriers to breast screening, particularly for Arabic-speaking women.

## 1. Introduction

Breast cancer is the most commonly diagnosed cancer for women globally and the second leading cause of cancer mortality for Australian women [1,2]. Breast screening programs provide an opportunity for early breast cancer detection and contribute to increased survivorship and reduced breast cancer mortality [3,4]. While controversy exists regarding the effectiveness of mammography [5,6], women need to be adequately informed about the benefits of screening alongside the potential harm of false positive results and unnecessary treatment when deciding to participate in breast screening [7,8].

The National BreastScreen Australia Program, a population-based breast cancer screening program, invites women aged 50 to 74 to have a free mammogram every two years [9]. The program is widely promoted by delegated state and territory breast cancer screen promotion organisations [10], and eligible women receive invitation and reminder letters at the address registered on the electoral roll. Letters have been found to be highly effective in engaging women in screening [11]. However, despite significant promotional efforts to increase screening participation, the Australian Institute of Health and Welfare reported in 2016 that Australia-wide breast screening participation rates had not increased from 54% since 2010 [12]. A significant proportion of eligible women remain under-screened and an exploration of potential determinants is warranted, including whether targeted approaches are required for specific cultural groups.

A variety of emotional, knowledge, and structural factors have been identified as barriers to women’s participation in breast cancer screening [13,14]. These barriers may be exacerbated for women from culturally and linguistically diverse (CALD) backgrounds who may have English language limitations or cultural and family factors that could impact upon their decision to screen [15].

Emotional barriers include anxiety driven by personal beliefs [13,14] and negative expectations, such as concerns related to perceived disrespect from screening providers and feelings of embarrassment [16]. Physical discomfort during previous mammograms has also been associated with under-screening [17]. Knowledge barriers include a lack of breast cancer risk awareness, not understanding the importance of screening regardless of current symptoms, and believing misinformation, such as ‘screening is harmful’ or that ‘there is no cure for breast cancer’ [18,19]. Structural barriers refer to practical challenges associated with making appointments, accessing screening services, and managing priorities, such as family commitments [16]. Financial barriers include direct costs of screening, and non-medical out of pocket costs associated with screening participation. While cost is often significant barrier to participation in screening elsewhere [20,21,22], direct costs are not a barrier to screening for Australian women given that the Australian program is free of charge.

Health literacy may provide a framework to better understand a woman’s vulnerability to breast screening barriers and under-screening. Health literacy is defined as the personal characteristics and social resources needed for individuals and communities to access, understand, and use information and services to make decisions about health [23,24]. Health literacy includes the capacity to communicate, assert, and enact these decisions. Previous breast screening research that explored health literacy focused on reading, writing, and numeracy skills related to comprehension of health information (known as functional health literacy) and identified associations with non-participation in breast screening [21,25,26]. Individuals with low functional health literacy were more likely to hold fatalistic cancer attitudes, less likely to identify the purpose of cancer screening procedures, and less inclined to engage with information about health conditions that they do not have [27].

In recent years, measurement of health literacy has advanced and it is now recognised as a multidimensional concept [28]. Health literacy includes a wide range of skills and resources that people use to engage with information, healthcare providers and services (see Table 1) [29,30,31]. These broader concepts may present further insights into the barriers to breast cancer screening. Women without reliable breast cancer screening information or adequate support from healthcare professionals may be more vulnerable to emotional barriers such as embarrassment, discomfort, and fear; women with less ability to find, understand, and appraise health information may be more susceptible to knowledge-related screening barriers; and women who lack social support or the skills needed to navigate healthcare services may be more likely to encounter structural barriers to screening. This framework for understanding risk factors related to under-screening for breast cancer may reveal new insights into women’s health literacy challenges and offer opportunities to develop new interventions that better support their participation in breast screening. 

The aim of this study was to explore if women from CALD backgrounds with lower health literacy reported greater emotional, knowledge, or structural barriers that may inhibit their participation in breast cancer screening. A further aim was to identify if women from CALD backgrounds with lower health literacy were more likely to report under-screening than women with higher health literacy.

## 2. Materials and Methods

### 2.1. Study Design

This study applied a cross-sectional survey design and targeted English-, Arabic-, and Italian-speaking women living or working in North West Melbourne, Australia. In this region of Melbourne, approximately 3.0% of the population speak Arabic and 3.3% speak Italian [32]. Surveys were administered in English, Arabic, and Italian with the assistance of bilingual Community Engagement Officers.

### 2.2. Study Setting

North West Melbourne has been identified by the Department of Health and Human Services (Victoria) Under-Screened Program as having lower than acceptable screening rates. This paper reports on a baseline survey results of the Ophelia (optimise health literacy and access) BreastScreen study: a collaborative project with the Department of Health and Human Services (Victoria), BreastScreen Victoria and Deakin University. BreastScreen Victoria is a public program that provides screening services (mammography) via many clinics (private and public providers) across the State. The Ophelia BreastScreen study aims to develop evidence-based, tailored interventions founded on consumer and provider health literacy strengths and limitations to improve breast screening uptake among women with CALD backgrounds. An additional cohort was included (Aboriginal and Torres Strait Islander women), and these findings will be reported separately.

### 2.3. Ethical Approval

Data were collected from March 2016 to September 2016. This study was approved by Deakin University (DUHREC project ID 2015-317) and Melbourne Health (HREC/16/MH/24. Project ID 2016.44). Informed written, implied, or verbal consent was obtained from all participants.

### 2.4. Participant Recruitment

Purposive sampling was used to recruit English-, Arabic-, and Italian-speaking women, eligible for breast screening between the ages of 50 to 74, living or working in North West Melbourne, Australia. Participant recruitment was guided by a multicultural Project Advisory Committee with representatives across community and service organisations. Invitations to participate were distributed through cultural social clubs and organisations, community centres, neighbourhood houses, community events, local radio, local newspapers, social media, and large employers of women in the target groups.

Paper-based surveys were delivered face-to-face in community settings (self-administered or with assistance from a Community Engagement Officer) and disseminated through community groups (a postage-paid envelope was provided for return of the survey). An online survey was promoted via project partners and community organisations. Computer assisted telephone interviews (CATI) were conducted by Italian-speaking data collectors to support the recruitment of Italian women. Surveys were provided to participants in English, Italian, and Arabic.

### 2.5. Measures

The Health Literacy Questionnaire (HLQ) is a widely-used multidimensional health literacy assessment tool for research and health service improvement [31]. The HLQ measures nine domains of health literacy, identifying strengths and challenges related to engagement with health information and services (see Table 1). The HLQ consists of 44 items across nine scales, with each scale containing four to six items. A four-point response scale is used to assess domains 1 to 5 (strongly disagree to strongly agree), and a five-point scale is used to assess domains 6 to 9 (cannot do or always difficult to always easy). The HLQ has a strong and reproducible theoretical structure that has been found to be robust in several studies [33,34,35,36]. The HLQ was translated using a formal forward and (blind) back translation procedure, with the forward translators guided by a detailed item intent document.

After defining the term ‘mammogram’, four survey items measured self-reported breast screening participation. These included “have you ever had a mammogram (or breast screen) to check for breast cancer?” and “if you have had a mammogram (or breast screen), when was your last one? (0–1 years, 2–3 years, 4–5 years, 6+ years, I have never had a mammogram)”. Further items enquired if participants recalled receiving a screening invitation letter from BreastScreen Victoria, and if the location of their last mammogram was at a BreastScreen Victoria clinic or at another private health service.

Eleven breast screening survey statements were used to assess emotional, knowledge, and structural barriers to breast screening. A broad list of statements was assembled from a review of publications describing barriers to breast screening [16,37,38]. From these, a summary list of statements considered to reflect barriers for women from CALD backgrounds was selected (see Table 2). Agreement with each breast screening belief statement was assessed using a five-point Likert scale ranging from strongly agree to strongly disagree. For the analysis of all breast screening survey statements, strongly agree and agree responses were assigned a value of “1”. Strongly disagree and disagree responses were assigned a value of “0”. Recoded responses for each type of breast screening barrier were summed together to produce counts of the three types of breast screening barriers.

The survey included demographic questions covering date of birth, living circumstances, country of birth, home postcode, language spoken at home, self-assessed English proficiency, educational attainment, and current employment status. Health status questions included the presence of chronic health issues and self-rated overall health (from 1, poor, to 5, excellent).

### 2.6. Analysis

Univariate comparisons of continuous variables between groups were conducted using Welch’s analysis of variance (ANOVA) and Games–Howell post-hoc tests to avoid the assumption of equal variances and sample size. Comparisons of categorical variables were conducted across the three language groups using cross-tabulations. Chi-square results were evaluated using *p*-values (*p* < 0.05) and adjusted standardised residuals (>1.96). For cross-tabulations with expected cell sizes of less than five, Fisher’s exact test was used.

Multiple Poisson regression (with log link function) was used to assess associations with the three types of breast screening barriers. Multiple logistic regression was used to assess associations with participation in breast screening. Age, completion of secondary education, language group, English ability, number of comorbidities, and the nine domains of the HLQ were controlled for in all multiple regression analyses. The multiple logistic regression also included counts for the three types of breast screening barriers as covariates. Regression results are presented with 95% confidence intervals (CIs). All statistical procedures were conducted using the R language and environment for statistical computing [39].

## 3. Results

### 3.1. Participants

In total, 377 surveys were completed. Women who reported a previous breast cancer diagnosis (*n* = 29), were aged 75 or over (*n* = 22), or who did not report their breast screening attendance (*n* = 9) were excluded from analyses. Data from 317 women were analysed. This total sample consisted of three cultural groups, defined by whether English (*n* = 105), Arabic (*n* = 60), or Italian (*n* = 152) was spoken at home.

Table 3 highlights demographic differences between the cultural groups. Compared to English- and Italian-speaking women, fewer Arabic-speaking women reported that they were currently employed or able to speak English very well. Arabic-speaking women also reported lower self-rated overall health and more chronic health conditions, including arthritis, diabetes, and cardiovascular problems. None of the Arabic-speaking women were born in Australia. Italian-speaking women reported a higher average age than the English-speaking women, and a lower proportion reported attaining a university-level education.

### 3.2. Screening Participation

Just over two thirds of women in the sample reported participating in breast screening within the past two years (Table 3). A greater proportion of Arabic-speaking women reported either under-screening or never screening. No significant differences in screening participation were found between English- and Italian-speaking women. A greater proportion of women who had under-screened or never screened also reported not receiving a screening invitation.

### 3.3. HLQ Scales and Barriers to Breast Screening

Table 4 demonstrates that, on average, Arabic-speaking women reported significantly greater difficulty for HLQ scale 7, *navigating the healthcare system*, scale 8, *finding good health information*, and scale 9, *understanding health information well enough to know what to do*, compared to English- and Italian-speaking women. Arabic-speaking women also reported a significantly greater number of emotional and structural barriers to breast screening (Table 4).

### 3.4. HLQ Scale Associations with Barriers to Breast Screening

Multiple Poisson regression results in Table 5 highlight significant associations between the HLQ scales and barriers to breast screening. A lower number of emotional breast screening barriers were reported by women who had higher scores for scale 1, *feeling understood and supported by healthcare providers*, 4, *social support for health*, and 9, *Understanding health information well enough to know what to do*. A lower number of knowledge barriers were reported by women who had higher scores for scale 1, *feeling understood and supported by healthcare providers*. A greater number of structural barriers were reported by women who had higher scores for scale 5, *appraisal of health information*.

Differences between cultural groups were also present. Compared with English and Italian-speaking women, Arabic-speaking women reported a 94% higher number of emotional barriers and an 85% higher number of structural barriers. Italian-speaking women reported a lower number of emotional barriers. Women who reported receiving a screening invitation letter had a lower number of emotional barriers. Additionally, women with greater comorbidities reported greater number of emotional barriers.

### 3.5. HLQ Scale Associations with Up-To-Date Breast Screening Participation

Results from logistic regression analyses evaluating associations with up-to-date breast cancer screening participation are presented in Table 5. Overall, no significant associations between health literacy and up-to-date breast screening participation were identified across all unadjusted and multivariate results. Results revealed that up-to-date screening was positively associated with receiving a screening invitation and negatively associated with emotional screening barriers. Receiving a screening invitation increased the odds of up-to-date screening by 246% (OR = 3.46, 95% CI = 1.81, 6.67), when all other demographic variables, HLQ scales, and barriers to breast screening were held constant. Each additional reported emotional barrier decreased the odds of up-to-date screening by 28% (OR = 0.72, 95% CI = 0.54, 0.94), when other values were held constant.

## 4. Discussion

This study investigated potential barriers to breast screening in a multicultural Australian context. Overall, Arabic-speaking women reported greater health literacy challenges and lower up-to-date breast screening participation than both English- and Italian-speaking women. However, findings suggest no evidence of a direct association between the many dimensions of health literacy and screening participation. Previous research has identified negative relationships between functional health literacy and screening participation [21,25,26], but this is the first study to have examined associations between a wide range of robust measures of health literacy and participation in breast cancer screening. These findings may reflect differences in the research contexts because previous studies were conducted in the United States of America where there is no free, comprehensive national breast screening program [21,22].

Consistent with previous research, emotional barriers were the most important barriers for up-to-date breast screening participation [14,38,40]. As previously found in the context of Arabic-speaking women [41], knowledge barriers were not associated with screening participation for any cultural group. Compared with English- and Italian-speaking women, Arabic-speaking women reported more emotional and structural barriers to breast screening, even when considering whether or not they reported receiving a screening invitation. The lower screening participation reported by Arabic-speaking women is partially explained by reports of a higher number of emotional barriers and a lower proportion of screening invitations received. Furthermore, Arabic-speaking women reported a higher prevalence of arthritis, diabetes, depression, anxiety, and a greater number of comorbid conditions overall, which may have limited their mobility and access to breast screening facilities.

Four health literacy domains were significantly associated with the three types of breast screening barriers. Domain 1, *feeling understood and supported by healthcare providers*, was related to fewer emotional and knowledge barriers. Direct recommendations to attend breast screening by health care providers are effective in increasing breast screening attendance [42]; however, even just maintaining an ongoing relationship with one or more health care providers has been found to increase the likelihood of screening [43]. Domain 4, *social support for health,* was related to fewer emotional barriers. Previous research has identified social support as a contributor to breast screening participation [44].

Domain 5, *appraisal of health information,* was related to a greater number of structural barriers. This suggests that women who reported a greater tendency to think critically about the information they received and if it was right for them may also perceive greater obstacles to arranging and attending a breast screen appointment. Domain 9, *understanding health information well enough to know what to do,* was also related to lower emotional barriers. This ability enables women to interpret available information, potentially reducing their emotional concerns and overall anxiety about breast screening [13]. The availability of this information, in the form of a screening invitation letter, may also reduce emotional concerns.

Overall, findings from this study indicate that cultural groups were an important factor in predicting emotional screening barriers, structural screening barriers, and breast screening participation. The potential links between emotional, knowledge, and structural barriers with the health literacy abilities of using, understanding, and accessing health information and services were not supported in this sample and context. The structure of breast screening services and the minimal demands they place on women’s ability to access, understand, and use health information and services may reduce the impact of health literacy challenges on ongoing breast screening participation.

Screening invitations had a greater positive association with up-to-date screening participation than all other investigated factors, which is consistent with the findings from a systematic review [11]. However, invitation letters written in English may have been a barrier to women from non-English speaking backgrounds. Women unable to read screening invitations provided in English are unlikely to report that they received these invitations, and this may explain why a smaller proportion of Arabic-speaking women reported receiving an invitation. Screening invitations written in multiple languages may help non-English speaking women to make their first contact with breast screening providers. Additionally, the use of the electoral roll as a register for screening invitation will only be relevant to those women who are enrolled to vote. Arabic-speakers have been found to be less likely to enroll due to language barriers [45]. Furthermore, recent migrants may be more likely to change address and their language difficulties may result in postal addresses being misreported.

A strength of this study was that the participant sample was based on targeted recruitment with extensive support from community organisations. Bilingual resources and staff supported broad participant recruitment within each cultural group. Among the women included in the study sample, self-reported rates of up-to-date breast screening participation for English- and Italian-speaking women (73% and 75%, respectively) were higher than rates Australia-wide (54%); however, previous research has found self-reported breast screening participation in minority groups to be accurate [46]. The sample may not be representative of women from their respective communities. Given the purposive sampling frame used in this study, caution should be taken with extrapolating the findings to other regions or other cultural groups. Population-based research may be warranted to confirm the current findings and to support the development of interventions that seek to increase breast cancer screening among disadvantaged and migrant groups. In addition, breast screening survey statements were not based on a previously validated measure and no validation of self-reported screening rates were undertaken.

## 5. Conclusions

Emotional screening barriers were negatively associated with up-to-date breast screening participation. Screening invitations were positively associated with up-to-date breast screening participation. These factors explained differences in screening participation across the three cultural groups. However, Arabic-speaking women reported the most difficulty with all three of these factors. Women with lower health literacy were not more likely to under-screen. However, they did report more breast screening barriers than women with higher health literacy. Future research should explore the nature of emotional barriers, particularly for Arabic-speaking women, and how these might be overcome. Emotional barriers to breast cancer screening appear to be an important target for interventions to increase participation rates, particularly for Arabic-speaking women. For these interventions to be most effective, it is important that they are culturally-sensitive and designed collaboratively with community members and other stakeholders [47].

## Figures and Tables

**Table 1:** Health Literacy Questionnaire (HLQ) domains with high and low descriptors.

No.	Low Level of the Construct	High Level of the Construct
*1*	*Feeling understood and supported by healthcare providers*	
	People who are low on this domain are unable to engage with doctors and other healthcare providers. They do not have a regular healthcare provider and/or have difficulty trusting healthcare providers as a source of information and/or advice.	Has an established relationship with at least one healthcare provider who knows them well and who they trust to provide useful advice and information and to assist them to understand information and make decisions about their health.
*2*	*Having sufficient information to manage my health*	
	Feels that there are many gaps in their knowledge and that they do not have the information they need to live with and manage their health concerns.	Feels confident that they have all the information that they need to live with and manage their condition and to make decisions.
*3*	*Actively managing my health*	
	People with low levels do not see their health as their responsibility, they are not engaged in their healthcare and regard healthcare as something that is done to them.	Recognise the importance and are able to take responsibility for their own health. They proactively engage in their own care and make their own decisions about their health. They make health a priority.
*4*	*Social support for health*	
	Completely alone and unsupported for health.	A person’s social system provides them with all the support they want or need for health.
*5*	*Appraisal of health information*	
	No matter how hard they try, they cannot understand most health information and get confused when there is conflicting information.	Able to identify good information and reliable sources of information. They can resolve conflicting information by themselves or with help from others.
*6*	*Ability to actively engage with healthcare providers*	
	Are passive in their approach to healthcare, inactive i.e., they do not proactively seek or clarify information and advice and/or service options. They accept information without question. Unable to ask questions to get information or to clarify what they do not understand. They accept what is offered without seeking to ensure that it meets their needs. Feel unable to share concerns. The do not have a sense of agency in interactions with providers.	Is proactive about their health and feels in control in relationships with healthcare providers. Is able to seek advice from additional healthcare providers when necessary. They keep going until they get what they want. Empowered.
*7*	*Navigating the healthcare system*	
	Unable to advocate on their own behalf and unable to find someone who can help them use the healthcare system to address their health needs. Do not look beyond obvious resources and have a limited understanding of what is available and what they are entitled to.	Able to find out about services and supports so they get all their needs met. Able to advocate on their own behalf at the system and service level.
*8*	*Ability to find good health information*	
	Cannot access health information when required. Is dependent on others to offer information.	Is an ‘information explorer’. Actively uses a diverse range of sources to find information and is up to date.
*9*	*Understanding health information well enough to know what to do*	
	Has problems understanding any written health information or instructions about treatments or medications. Unable to read or write well enough to complete medical forms.	Is able to understand all written information (including numerical information) in relation to their health and able to write appropriately on forms where required.

Source: Osborne et al. (2013) [31].

**Table 2:** Breast screening belief statements.

*Emotional barriers to screening*
1. I would not want to know if I have cancer
2. I put off having a breast screen because I have had a bad experience, or people I know have had a bad experience
3. I put off having a breast screen because I’m worried it will be very painful or uncomfortable
4. I put off having a breast screen because I’m worried they might find cancer
5. I put off having a breast screen because it is embarrassing
6. I have an objection to mammograms
*Knowledge barriers to screening*
7. Breast cancer screening could reduce my chance of dying from breast cancer
8. Breast cancer can often be cured
9. It is recommended that women my age have a breast screen
*Structural barriers to screening*
10. It is easy to arrange a breast screen
11. It is too difficult for me to get to a breast screen clinic

**Table 3:** Participant demographics.

Demographics	All Women		Cultural group						Cross-Tabulation and ANOVA
			English		Arabic		Italian		
	*n*	%	*n*	%	*n*	%	*n*	%	
Number of participants	317	100%	105	33%	60	19%	152	48%	
Age (Mean, SD)	61.07	6.89	60.78	6.31	59.28	7.04	61.98	7.1	**F(2154.8) = 3.27, *p* = 0.041**
Born in Australia	144	45%	81	77%	0	0%	63	41%	**x2(2) = 93.153, *p* < 0.001**
Lives alone	50	16%	19	18%	12	20%	19	12%	x2(2) = 2.342, *p* = 0.31
Not currently employed	207	65%	56	53%	47	78%	104	68%	**x2(2) = 13.712, *p* < 0.001**
Has a healthcare card	150	47%	36	34%	46	77%	68	45%	**x2(2) = 27.125, *p* < 0.001**
*Education*									
Did not finish primary school	19	6%	0	0%	12	20%	7	5%	**x2(12) = 61.874, *p* < 0.001**
Finished primary school	34	11%	0	0%	13	22%	21	14%	
Finished some of secondary school	64	20%	24	23%	9	15%	31	20%	
Finished secondary school	65	21%	21	20%	6	10%	38	25%	
Certificate, diploma, or apprenticeship	52	16%	23	22%	6	10%	23	15%	
Undergraduate university	43	14%	18	17%	6	10%	19	12%	
Postgraduate university	39	12%	19	18%	7	12%	13	9%	
*Health*									
Number of comorbidities (Mean, SD)	1.24	0.9	1.15	0.83	1.63	1.29	1.15	0.7	**F(2132.1) = 3.87, *p* = 0.023**
Fair or poor self-rated health	90	28%	14	13%	40	67%	36	24%	**x2(2) = 56.602, *p* < 0.001**
Cardiovascular problems	22	7%	5	5%	8	13%	9	6%	x2(2) = 4.813, *p* = 0.090
Arthritis	96	30%	21	20%	28	47%	47	31%	**x2(2) = 12.916, *p* = 0.002**
Diabetes	39	12%	8	8%	15	25%	16	11%	**x2(2) = 11.545, *p* = 0.003**
Depression	37	12%	9	9%	14	23%	14	9%	**x2(2) = 9.787, *p* = 0.007**
Anxiety	35	11%	10	10%	12	20%	13	9%	**x2(2) = 6.107, *p* = 0.047**
*How well do you speak English?*									
Very well	221	70%	98	93%	14	23%	109	72%	**x2(6) = 118.631, *p* < 0.001**
Well	59	19%	5	5%	20	33%	34	22%	
Not well	28	9%	0	0%	19	32%	9	6%	
Not at all	6	2%	0	0%	6	10%	0	0%	
Received a breast screening invitation	248	78%	88	84%	39	65%	121	80%	**x2(4) = 11.213, *p* = 0.024**
*Breast screening participation*									
Screened within the past 2 years	219	69%	74	70%	29	48%	116	76%	**x2(4) = 17.731, *p* < 0.001**
Under-screened	75	24%	26	25%	22	37%	27	18%	
Never screened	23	7%	5	5%	9	15%	9	6%	

Note: Results in bold are significant, *p* < 0.05.

**Table 4:** Descriptive statistics for the HLQ scales and barriers to breast screening.

Item	All Women	Cultural Group			ANOVA
		English	Arabic	Italian	
	*n* = 317 (100%)	*n* = 105 (33%)	*n* = 60 (19%)	*n* = 152 (48%)	
*HLQ [scale range]*					
1. Feeling understood and supported by healthcare providers [1–4]	3.20 (0.51)	3.25 (0.54)	3.23 (0.51)	3.15 (0.48)	F(2150.8) = 1.15, *p* = 0.319
2. Having sufficient information to manage my health [1–4]	3.08 (0.46)	3.09 (0.45)	3.08 (0.54)	3.06 (0.42)	F(2143.5) = 0.12, *p* = 0.884
3. Actively managing my health [1–4]	3.04 (0.52)	3.03 (0.56)	3.03 (0.62)	3.04 (0.44)	F(2138.2) = 0.04, *p* = 0.958
4. Social support for health [1–4]	3.07 (0.52)	3.05 (0.55)	3.14 (0.63)	3.07 (0.45)	F(2136.1) = 0.45, *p* = 0.640
5. Appraisal of health information [1–4]	3.00 (0.46)	2.98 (0.46)	3.08 (0.52)	2.97 (0.43)	F(2144.5) = 0.98, *p* = 0.379
6. Ability to actively engage with healthcare providers [1–5]	3.87 (0.71)	3.89 (0.72)	3.86 (0.67)	3.87 (0.71)	F(2154.6) = 0.04, *p* = 0.964
7. Navigating the healthcare system [1–5]	3.71 (0.65)	3.80 (0.61)	3.45 (0.64)	3.75 (0.65)	**F(2152.5) = 6.03, *p* = 0.003**
8. Ability to find good health information [1–5]	3.71 (0.70)	3.88 (0.59)	3.39 (0.77)	3.71 (0.71)	**F(2147.8) = 9.04, *p* < 0.001**
9. Understanding health information well enough to know what to do [1–5]	3.86 (0.69)	4.09 (0.59)	3.52 (0.72)	3.83 (0.69)	**F(2149.1) = 14.43, *p* < 0.001**
*Barriers to breast screening [count range]*					
Emotional [0–6]	0.51 (1.07)	0.46 (0.99)	1.25 (1.59)	0.25 (0.67)	**F(2124.1) = 11.83, *p* < 0.001**
Knowledge [0–3]	0.13 (0.41)	0.11 (0.42)	0.20 (0.55)	0.11 (0.34)	F(2134.3) = 0.69, *p* = 0.502
Structural [0–2]	0.19 (0.45)	0.10 (0.34)	0.50 (0.68)	0.12 (0.34)	**F(2133.5) = 9.28, *p* < 0.001**

Note: Results in bold are significant, *p* < 0.05; ANOVA = Analysis of Variance.

**Table 5:** Association between health literacy, barriers to breast screening and being up-to-date with breast screening participation.

	Barriers to Breast Screening			Up-To-Date Breast Screening Participation	
	Emotional	Knowledge	Structural		
	Poisson Regression			Logistic Regression	
	Score Ratio [95% CI]			Unadjusted OR [95% CI]	Adjusted OR [95% CI]
[Intercept]					0.15 [0.00–8.36]
Age	1.00 [0.97–1.02]	1.01 [0.97–1.06]	0.98 [0.95–1.02]	**1.07 [1.03–1.11]**	1.04 [0.99–1.10]
Completed secondary education	1.15 [0.78–1.72]	1.43 [0.76–2.75]	0.77 [0.47–1.27]	0.86 [0.52–1.42]	0.88 [0.42–1.85]
Arabic-speaking	**1.94 [1.24–3.04]**	1.21 [0.55–2.62]	**1.85 [1.06–3.27]**	**0.33 [0.18–0.59]**	0.60 [0.24–1.50]
Italian-speaking	**0.60 [0.39–0.91]**	0.71 [0.39–1.31]	0.65 [0.38–1.10]	**1.94 [1.19–3.18]**	1.10 [0.56–2.13]
Reported receiving a breast screening invitation	**0.55 [0.39–0.77]**	0.66 [0.38–1.17]	0.71 [0.47–1.09]	**3.84 [2.19–6.81]**	**3.46 [1.81–6.67]**
Number of comorbidities	**1.27 [1.12–1.43]**	0.97 [0.71–1.26]	1.11 [0.92–1.31]	**0.74 [0.57–0.96]**	0.80 [0.56–1.12]
*HLQ*					
1. Feeling understood and supported by healthcare providers	**0.50 [0.31–0.80]**	**0.43 [0.21–0.88]**	0.64 [0.36–1.15]	1.50 [0.93–2.43]	2.05 [0.90–4.79]
2. Having sufficient information to manage my health	1.15 [0.70–1.91]	1.74 [0.75–4.13]	1.61 [0.87–3.05]	1.28 [0.76–2.18]	1.04 [0.41–2.57]
3. Actively managing my health	0.92 [0.62–1.36]	1.12 [0.60–2.11]	0.74 [0.46–1.21]	1.43 [0.90–2.29]	1.19 [0.58–2.48]
4. Social support for health	**0.60 [0.40–0.92]**	1.16 [0.59–2.35]	1.04 [0.61–1.80]	1.31 [0.83–2.09]	1.18 [0.53–2.59]
5. Appraisal of health information	1.28 [0.77–2.13]	0.88 [0.39–1.98]	**2.44 [1.31–4.63]**	0.86 [0.51–1.46]	0.55 [0.21–1.35]
6. Ability to actively engage with healthcare providers	1.15 [0.78–1.72]	0.98 [0.53–1.84]	0.96 [0.59–1.56]	1.19 [0.84–1.66]	1.19 [0.55–2.63]
7. Navigating the healthcare system	1.35 [0.85–2.17]	0.90 [0.43–1.89]	1.25 [0.72–2.20]	1.10 [0.75–1.59]	0.78 [0.32–1.85]
8. Ability to find good health information	1.28 [0.81–2.07]	1.16 [0.58–2.40]	0.97 [0.57–1.64]	1.03 [0.73–1.45]	1.10 [0.47–2.60]
9. Understanding health information well enough to know what to do	**0.57 [0.38–0.86]**	0.75 [0.39–1.43]	0.64 [0.39–1.05]	1.03 [0.72–1.46]	0.63 [0.29–1.35]
*Barriers to breast screening*					
Emotional				**0.59 [0.46–0.74]**	**0.72 [0.54–0.94]**
Knowledge				0.82 [0.48–1.47]	1.14 [0.58–2.26]
Structural				**0.42 [0.25–0.70]**	0.57 [0.30–1.09]

Note: Results in bold are significant, *p* < 0.05; OR = Odds ratio.
